# Diversity and Colonization Strategies of Endolithic Cyanobacteria in the Cold Mountain Desert of Pamir

**DOI:** 10.3390/microorganisms9010006

**Published:** 2020-12-22

**Authors:** Nataliia Khomutovska, Asunción de los Ríos, Iwona Jasser

**Affiliations:** 1 Department of Ecology and Environmental Conservation, Faculty of Biology, Biological and Chemical Research Centre, University of Warsaw, Żwirki i Wigury 101, 02-089 Warsaw, Poland; jasser.iwona@biol.uw.edu.pl; 2 Department of Biogeochemistry and Microbial Ecology, The National Museum of Natural Sciences-CSIC, 28006 Madrid, Spain; arios@mncn.csic.es

**Keywords:** lithobionts, endoliths, Eastern Pamir, V3–V4 hypervariable region of 16S rRNA gene, ASV (amplicon sequence variant), scanning electron microscopy

## Abstract

Microorganisms can survive in extreme environments and oligotrophic habitats thanks to their specific adaptive capacity. Due to its severe and contrasting climate conditions, the cold mountain desert in Eastern Pamir provides a unique environment for analyzing microbial adaptation mechanisms occurring within colonization of endolithic habitats. This study aims to investigate the composition and structure of endolithic microbial communities and analyze the interactions between microorganisms and colonized lithic substrates. Endolithic biofilms were examined using scanning electron microscopy in backscattered electron mode (SEM-BSE) and next-generation sequencing (NGS) applying amplicon sequence variants (ASVs) approach. The investigation of the V3–V4 region of 16S rRNA gene revealed that endolithic communities are dominated by Actinobacteria (26%), Proteobacteria (23%), and Cyanobacteria (11.4%). Cyanobacteria were represented by Oxyphotobacteria with a predominance of subclasses of Oscillatoriophycidae, Synechococcophycideae, and Nostocophycidae as well as the rarely occurring Sericytochromatia. The positive correlation between the contribution of the orders Synechococcales and Rhizobiales to community structure suggests that some functionally closed taxa of Cyanobacteria and Proteobacteria can complement each other, for example, in nitrogen fixation in endolithic communities. The endolithic communities occurring in Eastern Pamir were identified as complex systems whose composition and structure seem to be influenced by the architecture of microhabitats and related microenvironmental conditions.

## 1. Introduction

Microorganisms play a crucial role in biogeochemical processes occurring in desert ecosystems [1]. Despite limited nutrient and water availability, microorganisms can form diverse ecological niches in these ecosystems, among which the lithobiontic niche (organisms occupying lithic microhabitats) is of particular importance [2,3]. Lithobionts are the essential biotic element of terrestrial desert ecosystems [2,3,4,5,6,7]. There are six main classes of lithobionts: (i) epiliths (colonizing rock surface), (ii) chasmoendoliths (developing in the cracks and fissures), (iii) cryptoendolith (growing in the natural pore space of rocks), (iv) euendoliths (boring actively into the rock substratum), (v) hypoendoliths (colonizing the rock matrix on the underside of the rock) and (vi) hypoliths (occurring on the ventral side of the rock) [8,9]. The lithobionts use rocks as substratum for their growth; consequently, they are involved in rock transformation through biological rock weathering [10,11]. The terrestrial endolithic niches are inhabited by chasmoendoliths and cryptoendoliths [10,11,12], while euendoliths are also a common feature in marine environments [13]. Lithobiontic microorganisms have to be well adapted to multiple stressors [4,5,7,9]. On the other hand, lithic substrates provide protection to endolithic microbial communities by limiting some external environmental stressors in particular microhabitats [3]. In fact, the rock architecture of colonized microhabitats conditions specific adaptations of lithobiontic microorganisms and facilitates their survival [4,13].

Microorganisms can supply barren soil with nutrients such as carbon (autotrophs) and nitrogen (diazotrophs), which can be used by other organisms [1,14]. At high altitudes cold deserts are characterized by sparse vegetation and barren sandy soil, thus, the lithic niche is inhabited by autophototrophs and diazotrophs which can become essential biotic elements of that ecosystem. The areas of lithic substrate inhabited by living organisms are complex and dynamic systems composed of many functionally dependent biotic and abiotic elements [3,4]. The functional and taxonomic diversity and metabolic activity of the microorganisms are genetically determined.

Some microbial species are more adaptable and can colonize different habitats in a poor desert ecosystem, while others can subsist only in particular microniches; these can be seen as habitat specialists. Understanding the role of particular taxonomic and functional groups of microorganisms in limited niches could help find their application in biotechnology and medicine [12,15].

There are numerous studies on endolithic microbial communities from the cold desert of Antarctica [4,10,16,17], but only a few describing endoliths from other cold desert areas, such as the Tibetan plateau [18] and Eastern Pamir [6]. Being a part of Central Asia, the Pamir Mountains represent geomorphologically diverse habitats (niches) with contrasting climatic conditions [6,19,20]. Hence, the cold desert of Pamir is of great interest for geomicrobiological investigations in cold environments. Moreover, the results of the study concerning the toxicity of cyanobacteria from the microbial mats carried out in Pamir, revealed the diverse structure of cyanobacterial communities but limited toxin production [19,20] which is in a strong contrast to the results of studies on benthic communities from the Arctic and Antarctic [21].

Our preliminary study reported the diversity of endolithic microbial communities in granites, limestones, and gneiss [6], while those from the quartzites have not been investigated. For the quartzites, hypolithic colonization is typical [2,3,5,22,23]. However, endolithic colonization is a common phenomenon for the geologically diverse Eastern Pamir [6]. Thus, Pamir could be considered a unique place for microbial adaptation to harsh conditions because of the diversity of and contrasting macro- and micro-habitat conditions. The combination of molecular techniques and electron microscopy could help to comprehensively investigate the diversity of the microbial communities and the interactions between microorganisms and lithic substratum occurring in the endolithic niche.

This study was designed to assess the diversity of endolithic microbial communities inhabiting granites and quartzites from Eastern Pamir. The study is part of a wider project targeting taxonomic and functional diversity at multiple niches in the cold mountain desert of Pamir (Tajikistan) based on the V3–V4 hypervariable region of 16S rRNA gene which allows for a comparative examination of the microbial profile along multiple ecological niches in a high mountain cold desert ecosystem. The research aims of this study were to (1) investigate the composition and structure of endolithic microbial communities; (2) study the complexity of the investigated communities (microbial–microbial and microbial–mineral interactions); and (3) explore cyanobacterial diversity and estimate their potential roles in the functioning of the investigated communities.

## 2. Materials and Methods

### 2.1. Study Area and Field Work

The Pamir Mountains lie between the world’s highest mountain ranges, namely, the Himalayas, the Tian Shan, Kunlun, Hindu Kush, Karakoram, Suleiman, and Hindu Raj [24]. In terms of climate, Pamir Mountains can be divided into Western Pamir with is characterized by mild climatic conditions, and Eastern Pamir which has a harsher and more arid environment. Eastern Pamir is an elevated plateau with high peaks that pass into flat wide valleys and mountain meadows with altitudes about 4000 m a.s.l. Based on the region’s complex geology, the Pamir Mountains can be categorized into four geological zones: Northern Sediment Zone, Northern Crystalline Zone, Southern Sediment Zone, and Southern Crystalline Zone [25]. The northern section is dominated by metamorphic rocks, basalts, and sedimentary rocks (Precambrian and Paleozoic). The central, highly complex section (Rushan-Pshart-Zone), was characterized by Vanselow [25] as the continental slope of an oceanic rift basin. The southern section of the Pamir Mountains is dominated by ophiolitic rocks, characterized by metamorphic rocks with different types of gneiss and granites [25].

Samplings were performed in July 2017. The collected samples were stored in sterile plastic bags and were divided into 4 pieces for the following analysis: the first was powdered and used for DNA extraction; the second was prepared and analyzed under an optical and scanning electron microscope (SEM); the third was kept for isolation and cultivation of cyanobacteria; the fourth was frozen and stored in the freezer. Samples selected for research were collected from the sites which are characterized by rather similar physical and chemical parameters of the soil, humidity, altitude, and annual temperature.

**Figure 1:**
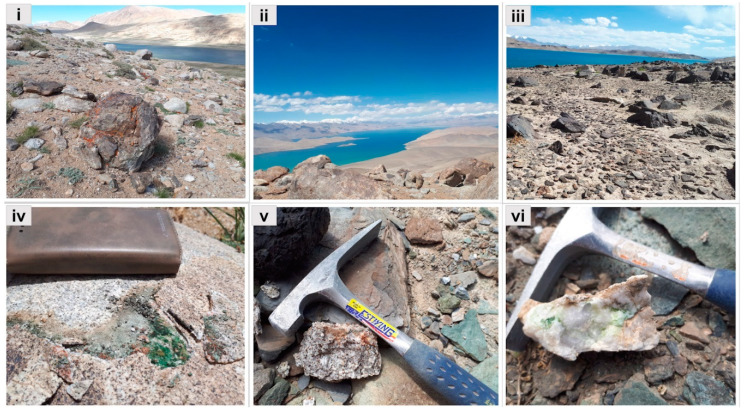
Sampling sites and samples representing the studied groups. (**i**) Sampling site and (**iv**) granite collected from the Lake Khargush, (**ii**) Lake Karakul, and (**v**) sample representing granite from group B, (**iii**) sampling site situated near Karakul, and (**vi**) sample of quartzites from group C.

Sample groups:

A). Granites collected from the slopes situated close to lakes Khargush (southern part of Eastern Pamir), and Rangkul (eastern part of Eastern Pamir): TAKHG and TARG.
B). Granites collected from sampling sites located nearby Lake Karakul (northern part of Eastern Pamir): TAKAG1, TAKAG2, and TAKAG3.
C). Quartzites collected from the same sampling area as the granites from group B which is nearby Lake Karakul: TAKAW1, TAKAW2, and TAKAW3 (Figure 1, Figure S1).

Most of the investigated samples were collected from mountainsides with a similar altitude (between 3901 and 4244 m a.s.l.) except for sample TAKAG1, which was collected from a sampling site located at 5019 m a.s.l. (Table S1)

### 2.2. Isolation, Cultivation, and Identification of Cyanobacteria

The rock pieces with green, blue-green, and brown visible colonization were implanted on Petri dishes containing WC or BG11 medium [26,27] solidified with 1.5–2% agar. The cultures were maintained in a plant growth chamber under the following conditions: 12 h/12 h light/dark regime with cool white light, at 16/18 °C.

### 2.3. DNA Extraction, PCR, Sequencing, and Sequences Processing

The powdered rock samples were treated with liquid nitrogen and the DNA was extracted using a Soil DNA Purification Kit (GeneMATRIX, EURx Ltd.; Gdańsk, Poland). The DNA extraction was conducted following manufacture protocols and was repeated at least three times for each sample. The concentration and quality of the DNA were checked with Hybrid Multi-Mode Reader (Synergy H1, BioTek, Bad Friedrichshall, Germany). The V3_V4_341f and V3_V4_785r primers [28] targeting the V3–V4 hypervariable region have been used for the amplification. The universal bacterial primers have been used in the present study to investigate cyanobacteria and other bacterial components of endolithic communities. The HotStarTaq DNA Polymerase (Qiagen, Hilden, Germany) was used to conduct the first PCR. The 25 mL of reaction mix containing 2.5 mL PCR Buffer, 5 mL Q-solution, 0.3 mL dNTP, 0.1 mL Taq, 0.5 µM of each primer, 15.1 mL nuclease-free water and 1 mL (5–65 ng) of DNA template has been prepared. The thermocycling conditions were as follows: 15 min of denaturation at 95 °C, followed by 25 cycles of 95 °C for 30 s, 55 °C for 30 s, 72 °C for 30 s, and final elongation at 72 °C for 10 min. The library preparation and sequencing were conducted commercially in the “BionanoPark” (Łódź, Poland). The mean number of reads based on the raw data was 54,755 obtained from the sequencing. After the chimeras’ removal and normalization, the mean number of reads per sample was 19,032. Sequences (2 × 300 bp) were paired in the QIIME2 environment (version 2020.2) [29]. The “demux” plugin was applied to check the quality of sequences. The “DADA2” plugin [30] (Divisive Amplicon Denoising Algorithm) was used to control the quality of demultiplexed reads. The ASVs-based (amplicon sequence variants) clustering method applied to group the sequences which were then classified using the SILVA database (release 132) [31]. The plugins and the parameters were used in the processing of the sequence are given in supplementary (Supplementary Table S3).

### 2.4. Validation of Cyanobacterial ASV

The V3–V4 sequences identified as cyanobacterial according to the SILVA [32] database have been verified using Cydrasil reference alignment and Cydrasil reference tree [33]. The sequences were aligned using the PaPaRa method [34], following the aligned sequences that were mapped applying the Evolutionary Placement Algorithm and the RAxML [35]. The raw sequences have been submitted to the NCBI database as a BioProject with ID PRJNA670585 and the submission ID SUB8336448.

### 2.5. Scanning Electron Microscopy in Backscattered Electron Mode (SEM-BSE)

Small fragments of rocks with endolithic colonization were prepared for observation under the scanning electron microscope (SEM) in backscattered electron mode (SEM-BSE) according to a procedure developed by Wierzchos and Ascaso [36] in the laboratory of Geomicrobiology and Microbial Ecology (MNCN-CSIC). Samples were first fixed with glutaraldehyde (3% *v*/*v*) and later with osmium tetroxide solutions (1% *w*/*v*), dehydrated in a graded ethanol series (from 30% to 100% *v*/*v*), and embedded in LR-White resin. Later, the resulting blocks of resin-embedded rock colonized samples were finely polished, carbon-coated, and observed using a FEI INSPECT 105 SEM microscope.

### 2.6. Statistical Analyses and Data Visualization

Hierarchical clustering analysis and non-metric multidimensional scaling (NMDS) have been conducted using the R environment [37]. The alpha diversity metrics were calculated in R Studio using “corrplot”, “vegan”, “factoextra”, “packages”, and “ggplot2”. The phylogenetic placement has been checked using iTOL [38]. The NMDS analysis was performed using the “vegan” package [39] and Bray-Curtis dissimilarity matrix.

## 3. Results

### 3.1. Morphology of Endolithic Biofilms

**Figure 2:**
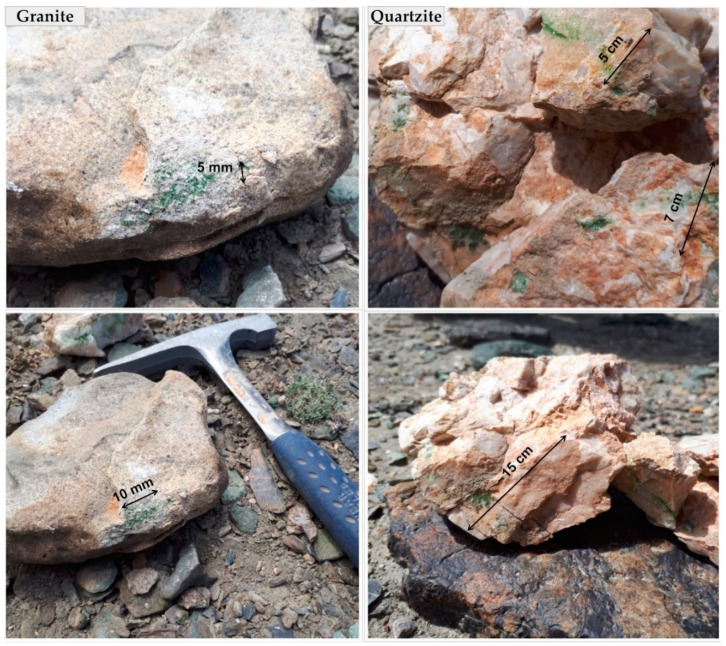
Endolithic biofilms occurring in granites and quartzites in Eastern Pamir. (Granite) the biofilms representing group A (TAKHG). (Quartzite) the samples representing group C (TAKAW1).

The endolithic communities from strongly weathered granites and less porous quartzites were rich in photoautotrophic microorganisms and were characterized by blue-green and green to brown colonization. Biofilms occurring in quartzites were smooth and softly attached to the substratum, while those from granites were mostly covered by tiny mineral particles (Figure 2). In the granites’ biofilms were located close to the dorsal surface of the rock, while in the quartzites they occurred both close to dorsal and ventral surfaces. The depth of substratum penetration also differed between quartzites and granites. Depending on the depths of the cracks, the endoliths inhabiting quartzites formed biofilms from a few millimeters up to several centimeters into the cracks of fractured quartzites.

Overall, the biofilms occurring in granites were characterized by brighter blue-green color, while those biofilms which developed in the cracks of the quartzites were of a darker yellowish or dark green to brown color. The presence of yellowish pigment in cyanobacterial sheaths was also observed under a light microscope during the examination of the biofilms.

### 3.2. Composition and Community Structure of Endolithic Microbial Communities

**Figure 3:**
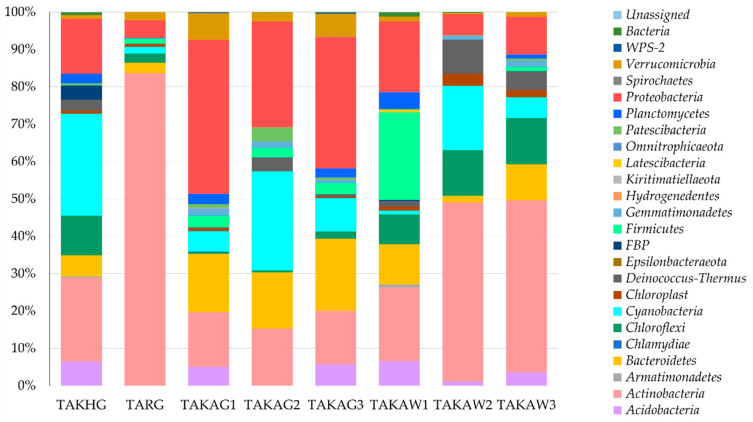
Structure of endolithic microbial communities at the phylum level based on the V3–V4 hypervariable region of 16S rRNA gene.

The results of amplicon-based analysis of Pamirian endoliths revealed that the communities were dominated by Actinobacteria (26% on average), Proteobacteria (23%), and Cyanobacteria (11.4%) including Sericytochromatia which were represented by 0.1% (47 reads) ASVs (amplicon sequence variants). Other bacterial phyla were less abundant, sharing about 39% altogether. The percentage of bacteria identified to phylum level only was 0.5% and unassigned 0.03% (Figure 3).

Actinobacteria were the principal contributors to the endolithic community in the TARG (group A) TAKAW2 and TAKAW3 samples (group C). Proteobacteria, Actinobacteria, and Bacteroidetes were abundant in the samples from group B and quartzites from group C (TAKAW1). The distribution of Oxyphotobacteria was irregular, with the highest percentage in samples TAKHG (27.3%) and TAKAG2 (26.6%). No statistically significant correlation was found between Cyanobacteria and other bacterial phyla.

**Figure 4:**
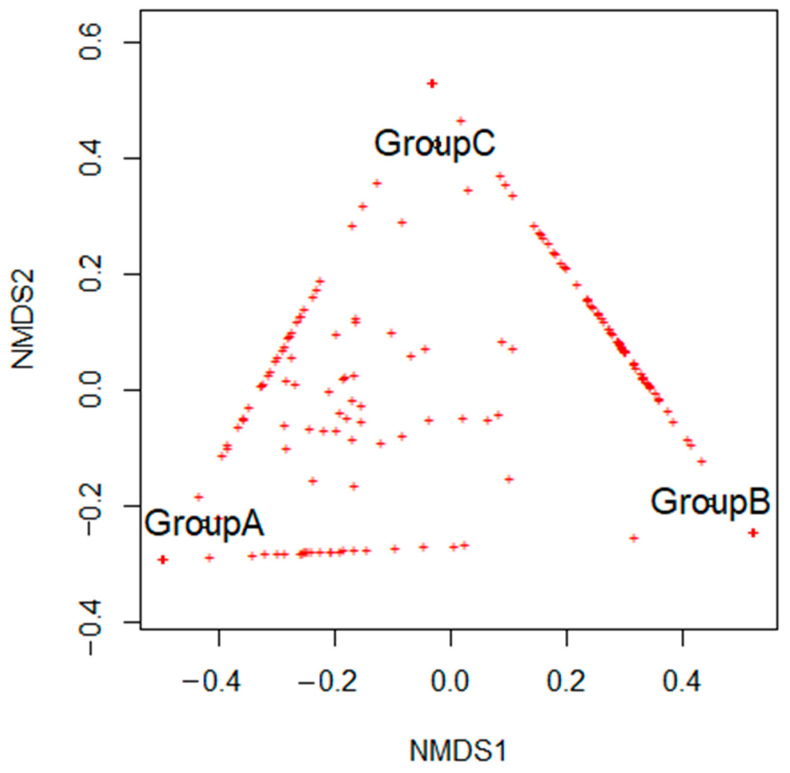
The non-metric multidimensional scaling (NMDS) analysis is based on the structure of microbial communities.

The results of non-metric multidimensional scaling, based on the structure of microbial communities, revealed that endolithic communities from studied groups were sharing only some ASVs, while most of the sequence variants seem to be group-specific. The NMDS plot showed a triangle-like arrangement of the communities’ structures. Thus beta-diversity analysis did not reveal a high resemblance between particular communities. (Figure 4).

The total number of representative sequences originating from chloroplast DNA was 587. Some of the sequences belonged to Chlorophyta, *Desmochloris halophila* (70 representative sequences), and *Picocystis salinarum* (11 representative sequences). Some sequences which could not be further classified were identified as “chloroplast” only.

Alpha diversity metrics calculated for studied communities demonstrate significant differences between the communities (Table 1). The Shannon diversity index has fluctuated from 4.25 (TARG, granite from group B) to 7.75 (TAKAW1, quartzite from group C). The phylogenetic diversity index (Faith PD) has oscillated between 11.39 (TAKAW2, group C) and 40.29 (TAKHG, group A). The numbers of observed ASVs varied significantly within the studied communities; the lowest value was observed for TARG (49) and the highest was identified for the community TAKHG (304), both of which belong to group A.

The clustering based on the alpha diversity revealed the presence of two main clusters of communities (Figure S2). Cluster 1 comprises more diverse endolithic communities (TAKAG1, TAKAG3, and TAKAW1), while cluster 2 comprises communities that were identified as less diverse (TAKAG2, TAKW2, and TAKAW3) (Figure S2). These clusters have the outliers in granite from Rangkul (TARG) and granite from Khargush (TAKHG). Communities from the first cluster are characterized by a high contribution of Actinobacteria and a lower percentage of Proteobacteria than the communities in the cluster 2. The second cluster was also characterized by stable contribution of Actinobacteria. The percentage of Cyanobacteria has fluctuated between the communities and was not characteristic of any of the clusters.

### 3.3. Diversity of Cyanobacteria and Association with Bacterial ASVs from Other Phyla

**Figure 5:**
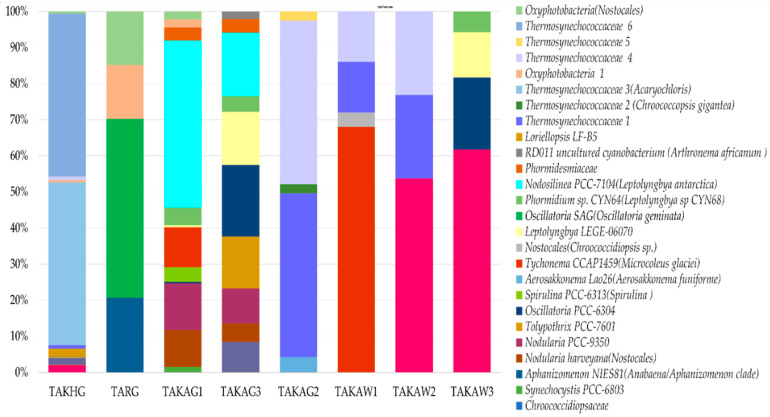
The V3–V4-based structure of endolithic cyanobacterial communities based on ASVs. The names of ASVs obtained using the phylogenetic placement method are given in brackets.

Cyanobacterial communities were represented by 26 groups of amplicon sequence variants (ASVs). The distribution between the communities was mosaic (Figure 5). The phylum Cyanobacteria were represented by Oxyphotobacteria (26 types of ASVs, 7444 representative sequences) and Sericytochromatia (one type of ASV, 47 representative sequences). Oxyphotobacteria comprises species from 3 classes of Oscillatoriophycidae (14 types of ASVs), Nostocophycidae, and Synechococcophycideae (6 types of ASVs each class). According to the SILVA database, endolithic communities of Oxyphotobcateria were represented by five orders: Oscillatoriales (seven types of ASVs), Nostocales (six types of ASVs), Synechococcales (six types of ASVs), Chroococcidiopsidales (five types of ASVs), and Spirulinales (one type of ASV) (Figure 5).

According to the SILVA database, the contribution of the order Synechococcales was high compared to those mainly consisting of species that can fix atmospheric nitrogen, such as Nostocales and Chroococcidiopsidales (Figure 5). The samples which were dominated by Synechococcales (TAKAG2 and TAKHG) (35% and 34% respectively) were also characterized by a greater contribution of order Rhizobiales (5% and 6% respectively) which belongs to Alphaproteobacteria. The Spearman correlation index calculated for the structure of Synechococcales and Rhizobiales is *r* = 0.76 (*p*-value = 0.03), however the correlation index was only calculated for eight studied communities here. The community TARG that was characterized by the absence of Synechococcales, but a greater contribution of Oscillatoriales and Nostocales according to the SILVA database contained also only 1% of Rhizobiales.

Analysis of cyanobacterial sequences placed on the Cydracil phylogenetic tree revealed that the sequences identified as *Thermosynechococcaceae* uncultured were located on the branch of *Chroococcopsis gigantea* with a like weight ratio of 0.9. The sequences of *Aliterella* CENA 595 according to the SILVA database were mapped on the branch of *Synechocystis* sp. with like weight ratio 1 (maximum). The results of the phylogenetic placement of the sequences with a low like weight ratio (about 18% with like weight ratio under 0.2) suggested the possibility of a novel taxa of endolithic cyanobacteria. Sequences belonging to one of the ASVs identified as “chloroplast” were placed on a branch of *Chlorogloea microcystoides* with a 0.83 weight like ratio.

The analysis of microbial association at the endolithic niche revealed the presence of four main clusters of endolithic communities (Figure S3). The biggest group (green clade) harbor filamentous cyanobacteria (including genera *Oscillatoria*, *Phormidium*, and *Leptolyngbya*) and different phyla with a high abundance of Proteobacteria (mainly represented by Gammaproteobacteria) and Bacteroidetes. In two smaller clades (red and blue clades) representatives of Oxyphotobacteria (classified as *Aliterella*, Thermosynechococcaceae, an Oxyphotobacteria) were associated with different genera of Actinobacteria and Alphaproteobacteria. Oxyphotobacteria (Cyanobacteria) from different orders were dispersed between the three biggest clades. The smallest clade (marked yellow in Figure S3) consisted of four ASV belonging to Actinobacteria, Gammaproteobacteria, and Alphaproteobacteria. The ASV identified as Thermosynechococcaceae grouped with Nocardioides (Actinobacteria). The *Aliterella* genus occurred in the clade with the genus *Rubrobacter* (including *Rubrobacter radiotolerans*) and Frankiales bacterium (Actinobacteria). Generally, communities were diverse and represented by different types of ASVs belonging to the same classes or phyla; therefore, a clear pattern of microbial associations has not been detected.

The results of 16S metabarcoding were compared with morphological analysis of isolated and cultured cyanobacteria in sampled communities.

**Figure 6:**
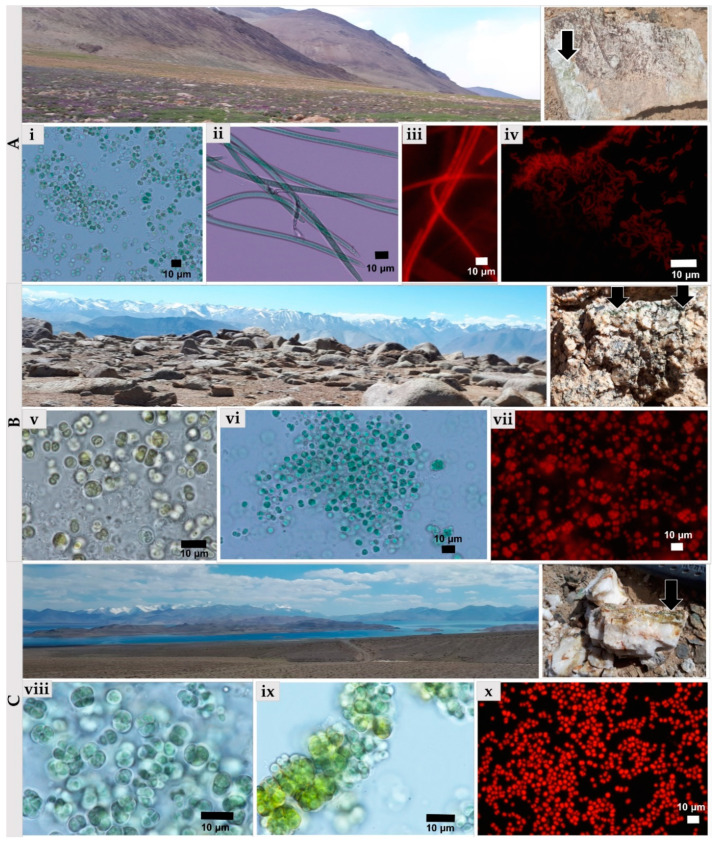
Isolates of endolithic cyanobacteria obtained from Pamirian granites and quartzites analyzed using light and epifluorescence microscopes. (**A**) Sampling sites and granitic rockfrom group A colonized by cyanobacteria (black arrow): (**i**) *Chroococcidiopsis*-like cyanobacterium, (**ii**) and (**iii**) *Microcoleus*-like morphotype, (**iv**) *Synechococcus*-like cyanobacterium. (**B**) the sampling sites and the granite from group B colonized by cyanobacteria (black arrow): (**v**) *Gloeocapsa*-like cyanobacterium, (**vi**) and (**vii**) *Chroococcidiopsis*-like cyanobacterium. (**C**) sampling sites and granitic rock from group C colonized by cyanobacteria (black arrow): (**viii**,**x**) *Chroococcidiopsis*-like cyanobacterium and (**ix**) *Aliterella*-like cyanobacterium.

The comprehensive analysis based on culture-dependent and culture-independent approaches revealed that *Chroococcidiopsis*-like taxa prevailed in cyanobacterial microbial communities in both types of mineral substratum, the granites, and quartzites. The granites were inhabited by more diverse cyanobacterial morphotypes than quartzites (Figure 6). 13 strains of cyanobacteria have been obtained and cultivated from the samples of Pamirian endoliths. *Chroococcidiopsis*-like cyanobacterium was present in most samples (TAKAW1, TAKAW2, TAKAW3, TAKAG1, and TAKAG3). Other less abundant morphotypes were *Microcoleus*-like (TAKHG and TAKAW1), *Synechococcus*-like (TAKHG and TAKAG2), *Gloeocapsa*- and *Phormidium*-like cyanobacteria (TARG), and *Cyanosarcina*-like cyanobacterium (TAKAHG) (Table S2. Comparative analysis of Pamirian endolithic communities using culture-dependent and culture-independent methods). The production of yellowish pigment during growth in laboratory conditions has been detected in the strains obtained from the quartzites (Figure 6ix).

### 3.4. Spatial Organization and Colonization Strategies Using SEM-BSE

**Figure 7:**
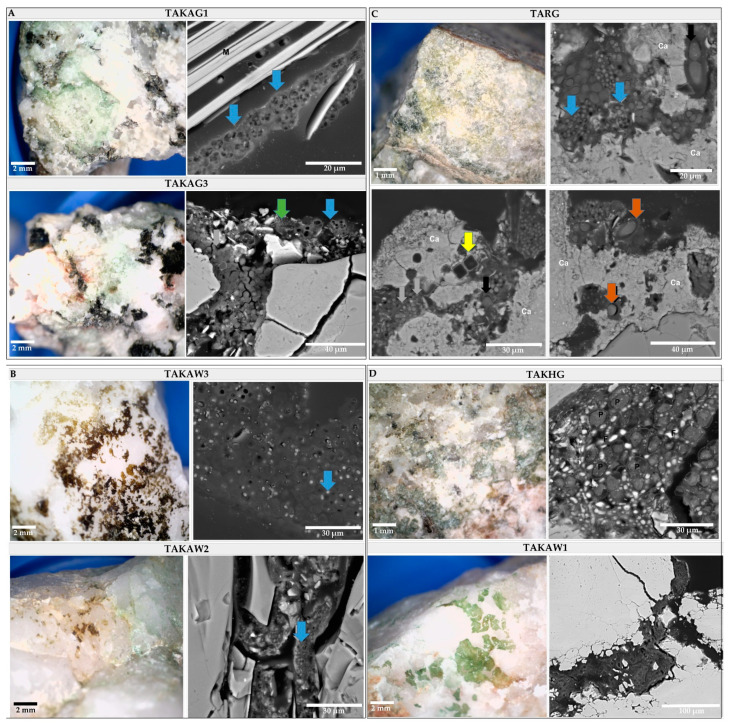
The top view of endolithic biofilms of the broken rocks and cross-sections of studied rock samples. (**A**) Cyanobacteria-dominated biofilm (TAKAG1 and TAKAG3) with coccoid cyanobacteria forming packets colonized mica (blue arrow) and smaller bacterial cells (green arrow). (**B**) Cryptoendolithic communities inhabiting quartzites are mainly represented by package-forming coccoid cyanobacteria (blue arrow). (**C**) Euendolithic community inhabiting Ca-rich layer (Ca), composed of different cyanobacterial morphotypes including coccoid (blue arrows) and filamentous, non-heterocystous cyanobacteria (brown arrows), and diatoms (yellow arrow). (**D**) Cross-sections of lichen-dominated biofilms (TAKHG, TAKAW1).

The study using the SEM-BSE technique revealed the presence of two types of microbial communities based on dominants: (1) “cyanobacteria-dominated” communities (Figure 7, TAKAG1 and TAKAG3), and (2) “lichen-dominated” communities (Figure 7, TAKHG and TAKAW1). Cyanobacteria-dominated biofilms were represented by two types of subcommunities: (i) dominated by aggregates of one type of coccoid cyanobacteria (Figure 7, TAKAG1, and TAKAG3), and (ii) biofilms harboring diverse cyanobacterial morphotypes (Figure 7, TARG).

The SEM-BSE analysis allowed the identification of different lithobionthic niches; three types of endolithic colonization are differentiated: chasmoendolithic (Figure 7, TAKAG1, TAKAG2, TAKAG3, and TAKHG), cryptoendolithic (Figure 7, TAKAW1, TAKAW2, and TAKAW3), and euendolithic (Figure 7, TARG). In granitic samples, cyanobacteria mainly colonized mica layers but also natural cracks and fissures, where they formed consortia with other bacteria of smaller size. In the euendolithic community cyanobacteria were represented by diverse morphotypes including coccoid, filamentous non-heterocystous, and filamentous heterocystous morphotypes (Figure 7, TARG). The chasmoendolithic communities occurring in quartzites consisted mainly of coccoid cyanobacteria which were characterized by small cell size and higher amounts of extracellular polymeric substances (EPS) in comparison to the microorganisms colonizing granites. In the granite sample, TARG, the colonization was associated with the presence of a layer rich in calcium (data not shown) indicating that sedimentary processes could have occurred at a previous stage of development of the parent rock. Based on 16S metagenomics analysis and morphology, the dominant cyanobacteria could correspond to genus *Aliterella* which belongs to Chroococcidiopsidaceae and was present in the samples TAKAW2 and TAKAW3.

Lichen-dominated communities colonized granite and quartzite substrates in two samples (Figure 7, TAKAW1, and TAKHG). Fungal components of lichen were observed occupying fissures and cavities at these samples and green-algae photobionts were detected through the 16S analysis. However, the taxonomic analysis of the photobiont has not been carried out yet.

## 4. Discussion

The results of the present study demonstrate that Pamirian endolithic microbial communities are heterogeneous in composition and spatial structure, which is only partially explained by the type of mineral substratum. The combination of microscopy and the 16S metabarcoding analysis demonstrated that the studied communities consisted of aggregates of diverse autotrophic and heterotrophic microorganisms which contribute to different ecological niches depending on the physicochemical properties of the colonized substrates. These results suggest that the contribution of the primary producers, nitrifiers, and denitrifiers and nitrogen fixers from different phyla, contrasted between studied communities, and the microorganisms within these communities were functionally closely interlinked. In this way, within an individual community, different functional and/or taxonomic groups could play a similar important role in primary production, nitrogen transformation, or rock weathering. Despite similarities in macro- and micro-habitat conditions of the analyzed lithic substrates, the structure of the communities was mosaic, which suggests that the functional composition and structure of the communities are also complex. The differences in the endolithic biofilm formation, colonization strategies, as well as the taxonomic structure of endolithic communities, can be explained by different physical–chemical and petrographic properties of colonized minerals substratum [40] and the capacities of adaptation of the involved microorganisms.

According to the SEM-BSE examinations, cyanobacteria and lichen symbionts were the main biomass-forming groups of microorganisms, just as it has been reported for Antarctic cold desert by de la Torre and co-authors (2003) [41], although other bacteria of lower size (including heterotrophic) were also abundant. The 16S-based analysis of the dominated phyla exposed the prevalence of Proteobacteria and Actinobacteria in these communities. Edaphic and lithobiontic microbial communities from cold deserts were characterized as rich in Actinobacteria and Cyanobacteria [15] which is compatible with some of our results.

The communities from group B granites were characterized by a similar contribution of Actinobacteria, Proteobacteria, and Bacteroidetes. However, these phyla were represented by the different families in individual samples. The Eastern mountains represents an environment rich in diverse rocks with different architecture and unique chemical properties that are colonized by cyanobacteria. We detected some similarities in the composition of Eastern Pamir communities to the endolithic communities from the cold desert of Antarctica [5,14,42], or endoliths from the high mountain desert of Tibet [18]. We also identified communities which were rather similar to the hot desert endoliths from the Atacama [43,44]. Additionally, we observed euendolithic communities which commonly occur in shallow and intertidal marine habitats and frequently occur in the carbonaceous and phosphatic mineral substrates [13]. These results prove the uniqueness of Eastern Pamir concerning the endolithic microorganisms. The differences in the methodology applied to the study of communities from different biogeographical regions, make it difficult to compare the structures of endolithic communities. Sequencing technology is developing all the time, and the recent study demonstrates a higher resolution of application of cyanobacteria-specific primers to analyze cyanobacterial communities [45]. However, the research presented here was designed to investigate cyanobacteria as a group but also to study their contribution to endolithic communities and other bacterial phyla. For this reason, universal bacterial primers were selected. The precise identification of endolithic cyanobacteria is difficult due to their scarce presence in sequence reference databases. Most of the closest relatives found for endolithic cyanobacteria during this study came from the environmental metagenomes, making the verification based on phylogenetic placement of the cyanobacterial sequences obtained in the present study difficult.

The granites, characterized by heterogeneous porosity, were colonized by more diverse cyanobacterial communities than quartzites, with similar physicochemical properties. Bioreceptivity, weathering processes, and the microhabitat architecture of rocks are significant factors in shaping the structure of endolithic communities [45,46]. The community structure of cryptoendolithic colonization occurring in quartzites, and of the chasmoendolithic colonization developed in granites, differed concerning dominated bacterial classes, indicating the influence of the ecological niche on community structure.

However, some similarities between the colonization of granites and quartzites were also found. Lichens and coccoid cyanobacteria were detected in both lithic substrates and the dominance of certain taxonomic groups were not associated with a specific substrate. The 16S-based structure of endolithic cyanobacterial communities demonstrated that the quartzites were dominated by specific ASVs, while the contribution of representative cyanobacterial taxa occurring in granite’s inhabiting cyanobacteria was more balanced. The dominance of aggregates of coccoid cyanobacteria in endolithic communities has been previously reported for hot deserts such as Atacama [12,44,45] or cold deserts as Antarctic Dry Valleys [17]. Interestingly, it seems that the heterogeneity of chemical composition of granitic rocks (e.g., occurrence of Ca-rich layer in the sample TARG) was related to appearance of the euendolithic colonization, which is atypical for terrestrial habitats. This euendolithic colonization demonstrated also a more diverse cyanobacterial community in terms of morphotypes than chasmoendolithic and cryptoendolithic communities occurring in other samples from Eastern Pamir. This again revealed the strong impact of physicochemical properties of the colonized lithic substrate on the diversity of endolithic communities [12]. Euendolithic colonization requires the active penetration of the substrate and consequently of specific abilities, suggesting that Pamirian endoliths have a broad genetic and metabolic potential and should be interesting for further studies on the diversity of functional genes. This phenomenon also demonstrates that chemical heterogeneity of the rocks in Eastern Pamir promotes higher diversity of endolithic bacteria in this region.

Contrasting differences and similarities of the composition and structure of the communities could be explained by the fluctuating chemical composition within the same type of rocks [2,9]. However, the differences detected in community structure found here cannot be explained only by the physicochemical properties of the colonized lithic substrate because these were found also between samples from the same type of rock (NMDS analysis). Because of the similarities in community structure found between samples from areas with different climatic conditions (e.g., samples from different altitudes and geographic location), we suggest that microenvironmental conditions also have a significant impact on the communities’ structure.

Slight differences detected in the color of studied biofilms developing in both types of mineral substratum could be considered as an adaptation to the colonization of rocks with different light regimes reaching the different microhabitats. The dark yellowish color of the biofilm TAKAW3 was detected by light microscopy, suggesting the presence of some accessory pigments in cyanobacterial sheaths. One of the UV-protecting metabolites in cyanobacterial sheaths is scytonemin. The production of this pigment was considered a strategy gained by lithobionths to cope with UV-radiation, especially for those occurring in more transparent types of rocks such as quartzites [45]. Similar observations have been reported for endoliths from regions with a high level of UV-radiation [45]. The fact that these cyanobacteria produce the yellowish pigment growing in medium featured by nitrogen limitation suggests that this secondary metabolite could result from stress-response. However, this observation has not been tested by high-performance liquid chromatography. Additionally, in the quartzite TAKAW3, the mucilage occupied a proportionally larger area in the endolithic biofilm than in granites.

Different biotic interactions are visualized by SEM-BSE concerning the microbial aggregates observed in endolithic habitats from Eastern Pamir. Microorganisms develop naturally in consortia in the environment, sharing some functions which are essential to survive in deserts or needed to cope with harsh conditions [46]. The investigation into relationships between the contribution of Cyanobacteria and Alphaproteobacteria in the communities revealed that the contribution of these two phyla into communities’ structure can relate to the importance of single taxa in the nitrogen transformation processes at the endolithic habitat. The taxa with similar functions belonging to different taxonomic groups can complement each other within the communities as was observed in the case of Synechococcales (Oxyphotobacteria) and Rhizobiales (Alphaproteobacteria). Rhizobiales are well known as plant-symbionts. Recently they have also been recognized as endosymbions of lichens, and [47]. However, the functional relationships between the orders of Oxyphotobacteria and Alphaproteobacteria is unexplored. This aspect of co-occurrence of Rhizobiales and Synechococcales should be studied further in future projects regarding their functional role in the endolithic communities. However, the analysis of the co-occurrence of microorganisms within the communities conducted at the ASV level did not reveal a clear pattern of microbial ASVs association belonging to particular taxonomic groups. The results are caused by a limited number of investigated samples (eight) one the one hand, and inefficiency of the 16S metabarcoding method applied to analyze microbial associations at the ASV, species, or even genus level on the other. These observations emphasize the presence of relationships between functionally closed taxa in endolithic communities and also emphasize the need of metagenomic analysis of the whole metagenomes in the study of poorly explored extreme environments such as Eastern Pamir and endolithic systems that occur in desert ecosystems [15,45].

Insight into the diversity and distribution of microbial taxa in extreme and in nutrient-limited environments, as well as an exploration of the relationships between abiotic and biotic components at different niches, could be key elements of understanding the functional diversity and the role of organisms in ecosystems [12,48]. Morphological and taxonomic diversity, as well as colonization strategies, can be established by a combination of 16S metabarcoding and SEM-BSE technique which give useful information to plan further analysis of functional genes. This complex approach has revealed, in the present study, that the structure of Eastern Pamir endolithic communities is only partially explained by the physicochemical properties of the colonized substrate. The composition and structure of these endolithic communities seem to be influenced by a variety of macro- and micro-environmental factors, such as water availability, nutrient status, microhabitat architecture, or microclimatic conditions. The structure demonstrates the variability of microbial consortia within the same type of substratum and point out the interconnection between taxa belonging to dominant groups of Bacteria (Proteobacteria, Actinobacteria, and Cyanobacteria) that are possibly complementing each other in the communities. Hence, these endolithic communities are complex and dynamic consortia, influenced by local and global environmental factors that make it difficult to predict the role of the taxonomic groups at particular habitats.

## Figures and Tables

**Table 1:** Alpha-diversity metrics calculated for studied endolithic communities.

Sample ID	Sample Group	Localization	Rock Type	Shannon ^1^	Observed ASVs	Pielou ^2^	PD ^3^
TAKHG	A	Khargush	granite	7.50	304	0.91	40.29
TARG	A	Rangkul	granite	4.25	49	0.76	11.49
TAKAG1	B	Karakul	granite	7.41	238	0.94	30.66
TAKAG2	B	Karakul	granite	5.52	82	0.87	15.49
TAKAG3	B	Karakul	granite	7.15	205	0.93	30.11
TAKAW1	C	Karakul	quartzite	7.75	269	0.96	33.12
TAKAW2	C	Karakul	quartzite	5.22	57	0.9	11.39
TAKAW3	C	Karakul	quartzite	6.00	95	0.91	17.52

**^1^** Shannon—Shannon diversity index, **^2^** Pielou—Pielou’s Evenness, **^3^** PD—Faith’s phylogenetic diversity.

## Supplementary Materials

The following are available online at https://www.mdpi.com/2076-2607/9/1/6/s1, Figure S1: The map of Tajikistan with the mapped sampling area. Modified map from WikiMedia (https://commons.wikimedia.org). Figure S2: Cluster plot based on an alpha-diversity matrix and the Euclidean distance, Figure S3. Co-occurrence of the most abundant ASVs of Oxyphotobacteria and other classes of Bacteria. Dendrogram based on Bray-Curtis dissimilarity coefficient. Table S1: Geographic localization of the sampling sites and environmental characteristics. Table S2. Comparative analysis of Pamirian endolithic communities using culture-dependent and culture-independent methods. Table S3. Scripts used for analyses of sequences in QIIME2 (version 2020.2). The tree is available online under the link https://itol.embl.de/tree/21287138665131573044368.
