# Supplementary material for: Diversity and Colonization Strategies of Endolithic Cyanobacteria in the Cold Mountain Desert of Pamir
Source: Microorganisms. 2020 Dec 22;9(1):6. doi: 10.3390/microorganisms9010006 (PMC7822004; doi:10.3390/microorganisms9010006)
Supplement: Supplementary file 1 [file microorganisms-09-00006-s001.pdf]

Table S1. Geographic localization of the sampling sites and environmental characteristics.

Sample ID	Group	Localization	Temperature[1]	EC[3]	Longitude	Latitude	Altitude[2]
TAKHG	A	Khargush	11.5 –15.4	195	$37^{0}26.455'$	$073^{0}04.521'$	4244
TARG	A	Rangkul	11.9 –12.1	218	$38^{0}26.009'$	$074^{0}17.429$	3901
TAKAG1	B	Karakul	9.7–13.2	201	$38^{0}50.660'$	$073^{0}23.644'$	5019
TAKAG2	B	Karakul	9.7–13.2	201	$39^{0}17.040'$	$073^{0}18.544'$	4022
TAKAG3	B	Karakul	9.7–13.2	201	$39^{0}17.046'$	$073^{0}18.519'$	4021
TAKAW1	C	Karakul	9.7–13.2	201	$39^{0}17.562'$	$073^{0}16.718$	4105
TAKAW2	C	Karakul	9.7–13.2	201	$39^{0}17.560'$	$073^{0}16.685'$	4111
TAKAW3	C	Karakul	9.7–13.2	201	$39^{0}17.035'$	$073^{0}18.483$	4025

[1]Temperature - summer average temperature [19]. [2] Altitude - m a.s.l. [3]EC - electrical conductivity of the soil in µS/cm^{-1}

Table S2. Comparative analysis of Pamirian endolithic communities using culture-dependent and culture-independent methods.

Sample ID	V3–V4 16S rDNA, Illumina MiSeq NGS (QIIME2, Silva 132)	Morphological identification of cultured cyanobacteria	SEM-BSE observations
TAKHG	*Aliterella*, *Chroococcidiopsis* sp., *Chroococcidiopsis, Crinalium,* Chroococcidiopsaceae, *Loriellopsis,* Thermosynechococcaceae, Oxyphotobacteria, "chloroplast"	*Cyanosarcina*-like cyanobacterium, *Microcoleus*-like cyanobacterium *Synechococcus*-like cyanobacterium.	Lichen, small bacterial cells
TARG	*Aphanizomenon, Oscillatoria,* Oxyphotobacteria "chloroplast"	*Gloeocapsa*-like cyanobacterium, *Phormidium*-like cyanobacterium	Oscillatoriales (*Phormidium*-like, *Nostoc*-like, *Gloeocapsa*-like cyanobacterium, diatoms
TAKAG1	*Synechocystis, Nodularia, Oscillatoria, Spirulina subsalsa, Tychonema, Leptolyngbya, Phormidium sp., Nodosilinea,* Phormidesmiaceae uncultured bacterium, Oxyphotobacteria "chloroplast"	*Chroococcidiopsis*-like cyanobacterium	*Gloeocapsa*-like cyanobacterial colony, small bacterial cells
TAKAG2	Aerosakkonema, *Thermosynechococcaceae* uncultured, *Thermosynechococcaceae*, "chloroplast"	*Synechococcus*-like cyanobacterium	Oscillatoriales cyanobacterium, *Synechococcus-* like cyanobacterium
TAKAG3	*Chroococcidiopsis, Nodularia harveyana, Nodularia, Tolypothrix, Oscillatoria, Leptolyngbya, Phormidium sp., Nodosilinea, Phormidesmiaceae, RD011* cyanobacterium, "chloroplast"	*Chroococcidiopsis*-like cyanobacterium, *Phormidium*-like cyanobacterium, *Nostoc*-like cyanobacterium	*Chroococcidiopsis* -like, *Nostoc*-like cyanobacterium, diatoms
TAKAW1	*Tychonema*, Thermosynechococcaceae uncultured, "chloroplast"	*Microcoleus*-like cyanobacterium	Lichen, small bacterial cells
TAKAW2	*Aliterella,* Thermosynechococcaceae uncultured, "chloroplast"	*Chroococcidiopsis* -like cyanobacterium	*Chroococcidiopsis*-like cyanobacterium
TAKAW3	*Aliterella, Oscillatoria, Leptolyngbya, Phormidium sp.,* "chloroplast"	*Chroococcidiopsis*-like cyanobacterium	*Chroococcidiopsis*-like colonies

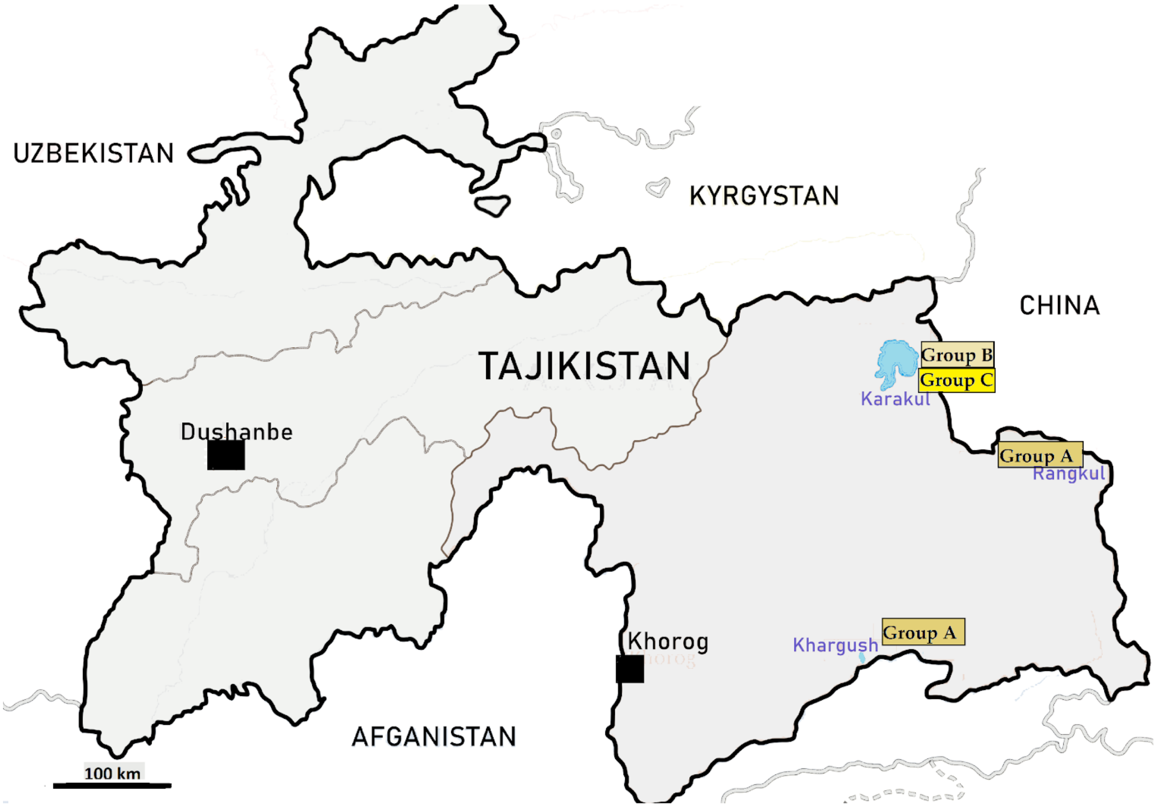

Figure S1. The map of Tajikistan with the mapped sampling area. Modified map from WikiMedia (https://commons.wikimedia.org).

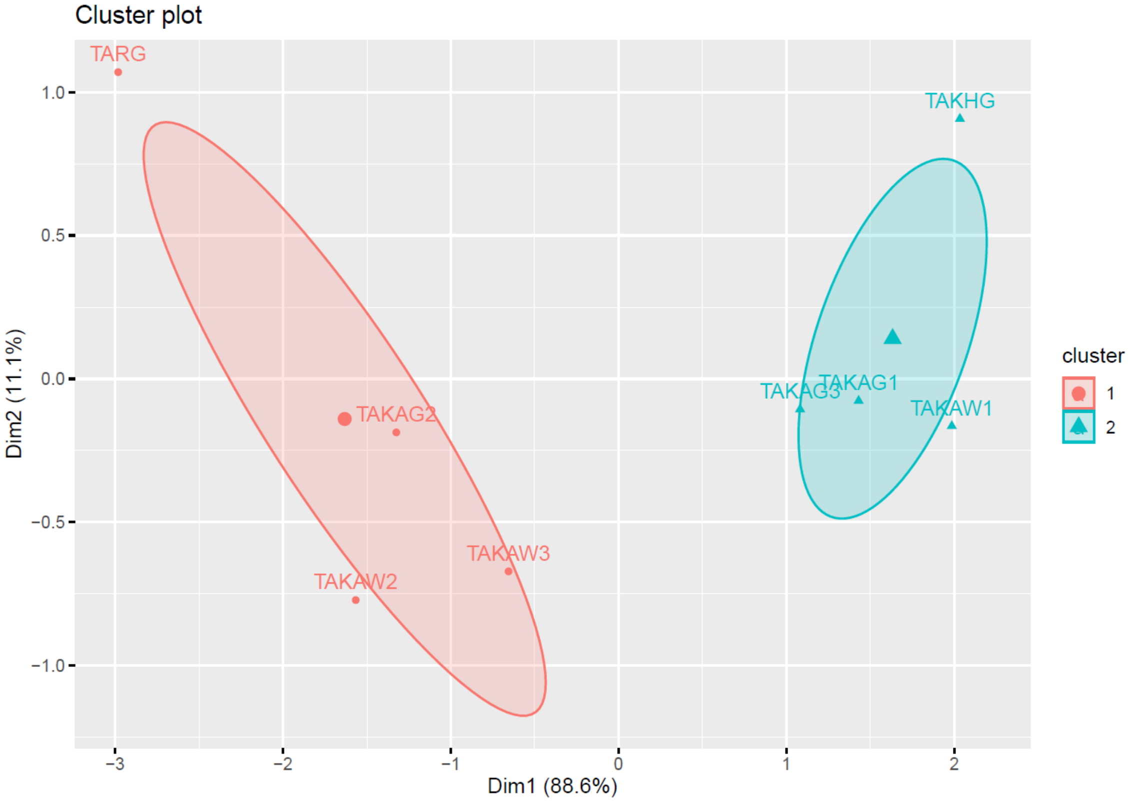

Figure S2. The cluster plot is based on an alpha-diversity matrix and the Euclidean distance.

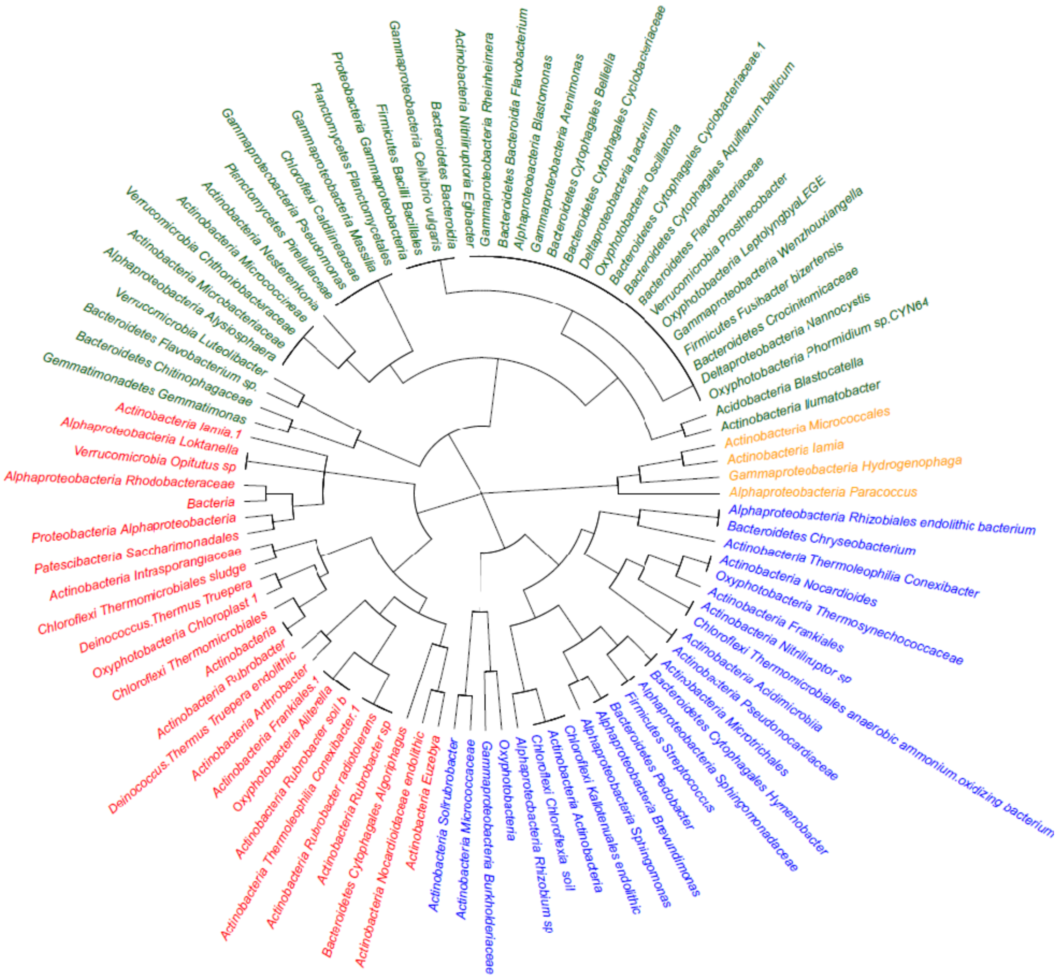

Figure S3. Co-occurrence of the most abundant ASVs of Oxyphotobacteria (Cyanobacteria) and other classes of Bacteria. The dendrogram based on Bray-Curtis dissimilarity coefficient.

Table S3. Scripts used for analyses of sequences in QIIME2 (version 2020.2)

```
# import data

qiime tools import

--type 'SampleData[PairedEndSequencesWithQuality]'

--input-path Rocks/

--input-format CasavaOneEightSingleLanePerSampleDirFmt

--output-path demux-paired-end.qza

# visualisation

qiime demux summarize

--i-data demux-paired-end.qza

--o-visualization demux-paired-end.qzv

# Denoise and dereplicate paired-end sequences. This method denoises paired-end
sequences, dereplicates them, and filters chimeras

qiime dada2 denoise-paired

--i-demultiplexed-seqs demux-paired-end.qza

--p-trim-left-f 5

--p-trim-left-r 5

--p-trunc-len-f 300

--p-trunc-len-r 300

--o-table table.qza

--o-representative-sequences rep-seqs.qza

--o-denoising-stats denoising-stats.qza


# Assign taxonomy using Silva 132 99% OTUs (full-length, seven-level taxonomy)
classifier with filtering sequences that are with less than 0.9 confidence

qiime feature-classifier classify-sklearn

--i-classifier silva-132-99-nb-classifier.qza

--i-reads rep-seqs.qza

--o-classification taxonomy.qza


#Filtering of Cyanobacterial sequences

qiime taxa filter-seqs

--i-sequences rep-seqs.qza

--i- taxonomy taxonomy.qza

--p-include Cyanobacteria

--o-filtered-sequences rep-seqs_Cyanobacteria
```

```
#Filter Cyanobacterial sequences
qiime taxa filter-seqs
--i-sequences rep-seqs.qza
--i-taxonomy taxonomy.qza
--p-include Cyanobacteria
--o-filtered-sequences rep-seqs_Cyanobacteria

# Assign taxonomy using Silva 132 99% OTUs
qiime feature-classifier classify-sklearn
--i-classifier silva-132-99-nb-classifier.qza
--i-reads rep-seqs_Cyanobacteria.qza
--o-classification taxonomy_Cyanobacteria.qza

# Merge taxonomy and table with numbers of futures
qiime taxa collapse
--i-table table.qza
--i-taxonomy taxonomy_Cyanobacteria.qza
--p-level 7
--o-collapsed-table collapsed_Cyanobacteria

#Convert biom file
biom convert
--input-fp feature-table biom
--o Collapsed_taxonomy.tsv
--to-tsv

#Visualize result
qiime taxa barplot
--i-table table.qza
--i-taxonomy taxonomy.qza
--o-visualization Viz_Seqs
```
